# High-fat diet-derived metabolite uridine diphosphate (UDP) impairs epithelial regeneration by P2Y_**6**_-receptor signaling

**DOI:** 10.1080/19490976.2026.2748160

**Published:** 2026-10-15

**Authors:** Rebecca Springer, Philip Claus, Marius Hörner, Christoph Otto, Natalie Burkard, Jakob Lauerer, Hanna Deinlein, Agnes Fekete, Werner Schmitz, Florian Seyfried, Nicolas Schlegel

**Affiliations:** a Department of General, Visceral, Transplantation, Vascular and Paediatric Surgery (Department of Surgery I), University Hospital Wuerzburg, Wuerzburg, Germany; b Pharmaceutical Biology, Julius-von-Sachs-Institute, Biocenter University of Wuerzburg, Wuerzburg, Germany; c Department of Biochemistry and Molecular Biology, Theodor Boveri Institute, Biocenter University of Wuerzburg, Wuerzburg, Germany

**Keywords:** Obesity, microbiota, intestinal barrier, junctional proteins, inflammation, TLR

## Abstract

Obesity is associated with a chronic low-grade inflammation affecting metabolic changes and organ dysfunction. Impairment of the intestinal epithelial barrier (IEB) and alterations in gut microbiota are considered to contribute to this phenotype by largely undefined mechanisms. Here, we used a diet-induced obesity model in rats to investigate the early interplay between a high-fat diet (HFD), gut microbiota composition, metabolic stool contents and IEB regeneration. Male Wistar rats received either a high-fat diet or a low-fat diet (LFD) ad libitum. HFD feeding resulted in weight gain and impaired oral glucose tolerance. Impairment of IEB function was evident by augmented lipopolysaccharide levels in the blood and reduced expression of junctional proteins Claudin-3, 4, and Desmoglein-2 in colonic samples of HFD rats. 16S-rRNA marker-gene sequencing of colonic microbiota revealed decreased phylogenetic diversity in HFD rats and significant alterations in microbial community composition. Detailed analysis revealed that content from HFD rats impaired intestinal epithelial regeneration in organoid assays. Metabolomic profiling identified uridine monophosphate/uridine diphosphate (UMP/UDP) metabolites as strongly enriched in HFD samples. UDP emerged as a key metabolite mediating impaired epithelial regeneration through P2Y_6_ receptor signaling, as pharmacological inhibition of P2Y_6_ with antagonist MRS2578 attenuated both UDP- and HFD-induced loss of epithelial regeneration. In summary, these data demonstrate that the diet-dependent stool metabolite UDP compromises intestinal epithelial repair via P2Y_6_ receptor signaling in early obesity. These findings link impaired epithelial regeneration to the onset of obesity-associated low-grade inflammation.

## Graphical abstract

**Figure:**
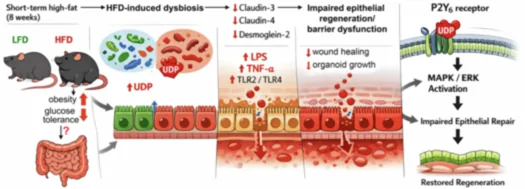

## Introduction

The World Health Organization has identified obesity as an epidemic, representing one of the most significant public health challenges of the 21st century. Obesity is associated with numerous serious health conditions, including type 2 diabetes, cardiovascular disease, and certain types of cancer, contributing to a substantial socio-economic burden on healthcare systems worldwide.^1^ ^,^ ^2^

Pre-clinical and clinical studies have demonstrated that obesity-induced metabolic disease and consecutive organ complications are caused by a chronic low-grade inflammation of adipose tissue.^3-5^ Increasing evidence indicates that the gut plays a central role in obesity-related metabolic dysfunction.^6-8^ A hallmark of obesity is the so called “leaky gut,” also referred to as impaired intestinal epithelial barrier (IEB) function. Levels of bacterial lipopolysaccharide (LPS) translocated from the gut lumen into the circulation represents a key marker of intestinal barrier dysfunction but also impaired regeneration in obese patients and animal models. Systemic bacterial LPS promotes chronic low-grade inflammation and is linked to obesity-related complications.^3^ ^,^ ^4^

The IEB is a crucial component of gut health, formed by a monolayer of specialized cells such as enterocytes, goblet cells, and Paneth cells.^9^ The paracellular space is sealed by junctional protein complexes including tight junctions, adherens junctions, and desmosomes.^10^ ^,^ ^11^ These proteins form a selective barrier that regulates nutrient absorption and protects against potentially harmful agents, such as ingested toxins or pathogenic microbes.^12^ It is important to note that epithelial turnover, differentiation and regeneration are essential to restore epithelial homeostasis. Meanwhile, it is well established that the intestinal microbiota contribute to the regulation of the intestinal epithelial integrity by largely undefined mechanisms and thereby are essential to maintain intestinal epithelial homeostasis.^3^ ^,^ ^8^ Alterations in the composition of the intestinal microbial community have been associated with high-caloric diets and obesity, implicating the microbiota as a critical mediator of obesity-related epithelial dysfunction.^13^

To address this, therapies aiming at stabilizing the IEB and overall epithelial regeneration, such as small molecule drugs^14^ and microbiota transplants,^15^ have shown promising results in reducing endotoxemia and improving metabolic health, offering potential strategies for managing obesity-related disorders. However, our understanding of the precise interaction between gut microbiota, microbial metabolites and the intestinal epithelium in the context remains limited. Especially, details on mechanisms are absent.

This prompted us to study how high-fat diet-induced obesity affects gut barrier integrity, epithelial regeneration, microbial composition, and gut metabolites in a diet-induced obesity (DIO) rat model. Due to the high microbial density and metabolic activity in the colon we focused on this section of the gut. We hypothesized that microbial-derived metabolites within the colonic lumen contribute to gut intestinal epithelial dysfunction and impaired regeneration by altering epithelial cell integrity and promoting inflammation. To address this, we employed both *in vivo* and *in vitro* approaches to explore the mechanistic links between microbial community composition, microbial metabolites, and intestinal epithelial dysfunction.

## Materials and methods

### Animal experiments

All animal experiments were carried out in accordance with the European Directive 2010/63/EU on the protection of animals used for scientific purposes and were approved by the local regulatory authorities, the Government of Lower Franconia, Bavaria, Germany (approval number: Az 2-1711). All procedures and findings are reported in accordance with the ARRIVE guidelines. Sample size calculation was based on expected differences in weight gain between different experimental groups. Male outbred Wistar rats (Wistar Crl:WI, *n* = 12) from Charles River, Sulzfeld, Germany, aged five to seven weeks and weighing 250-300 g were allocated to two groups. The LFD group (*n* = 6) received a standard chow diet (#1320M fortified, Altromin, Lage, Germany), while the HFD group (*n* = 6) received a high-fat diet (C 1090—60, Altromin) supplying 60% of energy from fat. Both groups were maintained on their respective diets for 8 weeks. Rats were housed in pairs in Makrolon type IV cages, enriched with environmental items, under controlled conditions 12-h light/dark cycle, 20 °C–24 °C and 45%–65% humidity. Body weight (BW) was measured daily to monitor weight gain and rat's health and well-being. Food intake (FI) was measured daily by weighing remaining food at the same time each day. Daily food consumption per cage was calculated based on the difference in food weight between the current day and the previous day. Treatment and measurement procedures were performed under standardized conditions and identically across experimental groups. No specific measures were taken to control for treatment order, measurement order, or cage location. No blinding was performed.

### Oral glucose tolerance tests (OGTT)

An oral glucose tolerance test (OGTT) was performed in week seven after starting diet feeding. To avoid oral gavage and minimize stress, animals were trained on two occasions to voluntarily drink 10 mL/kg body weight of a 25% glucose solution within 10 min. Rats were fasted for 8 h prior to the test, and blood samples were collected from the tail vein immediately before and at 15, 30, 60, 90, and 120 min after glucose ingestion. Blood glucose levels were measured using a GlucoSmart Swing glucometer (MSP Bodman GmbH, Bobingen, Germany), and the area under the curve (AUC) was calculated to quantify glucose tolerance.

### Blood and tissue collection

Animals were not fasted prior to sacrifice, in order to obtain the intestinal contents. At week eight of the experiment, animals were anaesthetized with 3% isoflurane (Iso-Vet, Dechra Veterinary products, Germany) in oxygen (1.6 l/min) and maintained at 1.5%–2.0% via facemask. Thirty minutes prior to surgery, animals received 2 mg/kg butorphanol (Butorgesic®, cp-pharma) subcutaneously (s.c.). The abdominal cavity was opened, and blood was collected from the portal vein. Additionally systemic blood was collected by cardiac puncture, resulting in euthanasia by exsanguination. Blood was centrifuged at 5000 rpm for 10 min, and serum was stored at −80 °C. Following this procedure, animals were euthanized by cardiac puncture. For microbial analysis and cell culture experiments, colonic contents were collected using a moist cotton swab, transferred to cryotubes, and snap-frozen in liquid nitrogen. Colonic segments were either snap-frozen and stored at −80 °C or fixed in formalin and embedded in paraffin. Some blood and tissue samples could not be included in individual analyses because of hemolysis, protein degradation, RNA degradation, or insufficient sample quality. The number of specimens included in the specific analysis are shown in the figure legends.

### Immunoassays

Serum LPS levels were measured using a Limulus Amebocyte Lysate Chromogenic Endpoint Assay (HIT302, HycultBiotech, Uden, Netherlands, 1:10 dilution) according to the manufacturer's instructions. Plasma levels of multiple cytokines were quantified using a multiplex bead-based immunoassay (MILLIPLEX MAP Rat Adipokine and MILLIPLEX MAP Rat Cytokine/Chemokine, Merck, Darmstadt, Germany).^16^ The adipokine panel measured leptin and PAI-1, while the cytokine panel measured IL-6, IL-10, IL-12, IL-13, IL-17A, IFN-γ, MCP-1, TNFα and CCL5. For the adipokine panel, serum was undiluted, for the cytokine panel, serum was diluted 1:5. Hemolytic blood samples could not be analyzed in the ELISA so that a total of 4 LFD and 5 HFD blood samples were included in these measurements.

### Caco2 cell culture

As reported previously,^17^ Caco2 cells (ATCC, Wesel, Germany) were cultured in Dulbecco's Modified Eagle's Medium (DMEM; Sigma-Aldrich, St. Louis, MO, USA) supplemented with 100 U/mL Penicillin-Streptomycin (Thermo Fisher) and 10% fetal calf serum (FCS; Thermo Fisher) as previously described.^17^ Cells were passaged every 7 d. For experiments, FCS was substituted for 10% colonic content.

### Murine organoid culture

Murine organoid generation was carried out as described before.^18^ ^,^ ^19^ For the current manuscript we used organoids generated from 3 individual mice. Excised tissue was placed in 20 mL ice-cold PBS (4 °C), vortexed briefly for 5 s, and the supernatant was removed. This washing procedure was repeated until no visible debris remained in the supernatant. Subsequently, the tissue was incubated in PBS supplemented with 2 mM ethylenediaminetetraacetic acid (EDTA; Sigma-Aldrich) for 30 mi under gentle rotation to facilitate crypt isolation. After EDTA treatment, samples were rinsed in PBS by carefully inverting the tube five times. The mucosal layer was then transferred into a fresh tube containing 10 mL PBS and manually shaken for 10 s; this step was performed twice using new tubes each time.

Crypt fractions were examined microscopically, and supernatants enriched for intact, viable crypts were combined and centrifuged at 350 × *g* at room temperature for 3 min. The resulting pellet was resuspended and washed in 10 mL of basal medium composed of Advanced DMEM/F12 (Thermo Fisher, Invitrogen) supplemented with 1 × N2, 2 × B27, 1 × Anti-Anti, 10 mM HEPES, and 2 mM GlutaMAX-I (all Thermo Fisher, Invitrogen), as well as 1 mM N-acetylcysteine (Sigma-Aldrich). Following centrifugation at 300 × *g* at room temperature for 3 min, the pellet was embedded in cold Growth Factor Reduced Basement Membrane Matrix (Corning, Corning, NY, USA).

Aliquots of 5–10 μL were plated as droplets in multiwell plates and allowed to polymerize by incubating the plate inverted at 37 °C for 20–30 min until the matrix solidified. Organoids were cultured in medium consisting of 50% fresh basal medium and 50% Wnt3A-conditioned medium, supplemented with the following additives: 500 ng/mL human recombinant R-Spondin 1 and 50 ng/mL human Epidermal Growth Factor (hEGF) (both Thermo Fisher, PeproTech); 100 ng/mL recombinant Noggin (PeproTech); 10 mM nicotinamide, 10 μM SB202190, and 10 nM [Leu15]-Gastrin I (Sigma-Aldrich); 500 nM A83-01 (Tocris Bioscience, Bristol, UK); and 500 nM LY2157299 (Axon MedChem, Groningen, Netherlands). Immediately after passaging, 10 μM Y27632 dihydrochloride (Tocris Bioscience) was added to the culture medium.

Growth patterns of organoids incubated with colonic content and/or different mediators as indicated below were continuously monitored using zenCELL owl live-cell imaging system (innoME GmbH, Espelkamp, Germany). Images were acquired over 72 h every 10 min while cultures were maintained at 37 °C and 5% CO₂. Organoid quantification was performed using a custom image analysis pipeline (Python 3.14). Brightfield time-lapse recordings (2588 × 1942 pixels, 0.464 μm/pixel, 10 min acquisition interval over 72 h) were sampled hourly for analysis. Segmentation combined two complementary approaches: Cellpose-SAM (v4.2.1) at two size scales to detect large, lumen-filled organoids, and a texture-based method using the local standard deviation (15-pixel window) to capture compact cell clusters. To ensure comparability across groups, the segmentation threshold was calibrated once across all series (Otsu's method on pooled texture values) and held constant for all images. Touching objects were separated by watershed transformation using h-maxima seeds. Individual organoids were tracked by area overlap between consecutive frames; image stability was verified beforehand by phase correlation (0 pixel displacement). Specific growth rates in μm were determined per organoid by linear regression of ln(area) against time up to the area maximum, with doubling time (h) was calculated as ln(2)/μ.^20-24^ Statistical differences in organoid growth parameters between groups were assessed using Kruskal–Wallis test followed by Dunn's test for multiple comparisons. Importantly, the unbiased analysis included all detected organoids, irrespective of whether they exhibited measurable growth during the observation period.

### Generation of tissue and 2D-organoid lysates

Intestinal tissues were processed immediately after dissection to isolate epithelial cells for western blot analysis. Tissue sections were sequentially incubated with DTT (5 mmol) and then EDTA (5 mmol) at 37 °C, with epithelial cells released by vortexing and collected from the supernatant. EDTA incubation was repeated for a total of three cycles. The collected cells were centrifuged, washed with PBS, and lysed in SDS buffer containing protease inhibitors. Cell lysates were finally sonicated prior to analysis. Frozen samples were homogenized in tissue lysis buffer, centrifuged for 10 min at 13 krpm and sonicated for 30 s. 2D organoid monolayers were incubated in cell recovery solution for 30 min and detached from the plate by vigorously pipetting up and down. The collected cells were centrifuged, the pellet was washed in ice cold PBS and suspended in 50 μl SDS lysis buffer.

### Western blot

SDS gel electrophoresis and blotting were performed after normalization of protein amount using the bicinchoninic acid (BCA) assay (Thermo Fisher, Waltham, MA, USA) as described previously.^25^ Primary and secondary antibodies were diluted as detailed in Table 1 in 5% milk and 0.1% Tween. Bound immunoglobulins were visualized by chemiluminescence (Thermo Fisher) and quantified by densitometry (ChemicDoc Touch Bio-Rad Laboratories, Hercules, CA, USA), normalized to a reference protein (β-actin), and expressed relative to the LFD group for rat samples.

**Table 1.** Antibodies and dilutions used in western blotting (WB) and immunofluorescent (IF) staining.

Antibody	Source	Reference no.	Dilution WB	Dilution IF
Claudin 3	Thermo Fisher	PA5-16867	1:500	n/a
Claudin 3	Sigma-Aldrich	SAB4500435	n/a	1:800
Claudin 4	Thermo Fisher	32-9400	1:500	1:1600
Desmoglein 2	Abcam	Ab150372	1:1000	n/a
Tumor necrosis factor alpha	Abcam	Ab183218	1:1000	n/a
IkB alpha	Cell signaling	4812S	1:1000	n/a
Phosphorylated IkB alpha	Cell signaling	2859	1:1000	n/a
goat-anti-rabbit Cy3	Dianova	111-165-003	n/a	1:600
DAPI	Calbiochem	268298	n/a	1:500
Ki67	Abcam	ab15580	n/a	1:200
Beta actin	Sigma	A3854	1:1000	n/a

### Immunofluorescence

Paraffin embedded tissues were sliced into 1 μm thick sections and mounted on microscopy slides. Immunostainings were carried out as described previously.^26^ Primary and secondary antibodies were diluted as detailed in Table 1 and incubated over night at 4 °C. Sections were mounted with Entellan (Merck) and coverslipped. Immunofluorescence images were captured using an AxioImager.M2 with ApoTome.2 (Zeiss, Oberkochen, Germany). Analysis of immunofluorescence intensities was performed with ImageJ by segmenting areas with a fluorescent signal and measuring signal intensity inside of these areas.

### RT-qPCR

Total RNA was isolated from organoid cultures and tissue samples using TRIzol reagent following established protocols.^25^ RNA yield and purity were assessed with a Nanodrop™ 2000C spectrophotometer (Thermo Fisher Scientific). For cDNA synthesis, 1 μg of RNA was reverse transcribed using the iScript cDNA Synthesis Kit (Bio-Rad) in a Primus 96 thermal cycler (Peqlab, Erlangen, Germany). Quantitative PCR analyses were performed with 100 ng of cDNA per reaction, run in duplicate, using iTaq Universal SYBR Green Supermix (Bio-Rad) together with 100 pmol of gene-specific forward and reverse primers (Eurofins, Hamburg, Germany) on a CFX96 Touch real-time PCR system (Bio-Rad). Amplification was carried out for 40 cycles with an annealing step at 60 °C and an extension step at 72 °C. Relative transcript levels were calculated using the ΔΔCt approach. Expression of target genes was normalized to the housekeeping gene glyceraldehyde-3-phosphate dehydrogenase (GAPDH) and presented relative to the LFD control group. Primer sequences are provided in Table 2.

**Table 2.** Primers used for qPCR.

Name of the gene	Nucleotide sequences (5′-3′)
Rat toll-like receptor 2 (Tlr2)	F-TACAGGGATACAGGCCGTCA
	R-CTTGGAGCCGAGGCAAAAAC
Rat toll-like receptor 4 (Tlr4)	F-CCGCTCTGGCATCATCTTCA
	R-TCCCACTCGAGGTAGGTGTT
Rat beta-2-microglubulin (B2M)	F-CCGTGATCTTTCTGGTGCTT
	R-ATTTGAGGTGGGTGGAACTG
Mouse toll-like receptor 2 (Tlr2)	F-GCCACCATTTCCACGGACT
	R-GGCTTCCTCTTGGCCTGG
Mouse toll-like receptor 4 (Tlr4)	F-GGCATGGCATGGCTTACACC
	R-TCTCCACAGCCACCAGATTCT
Mouse beta-2-microglobulin (B2M)	F-GGTGCTTGTCTCACTGACCGGC
	R-TTCTCCGGTGGGTGGCGTGA

### Transcriptome sequencing

Total RNA was isolated from organoids and tissue samples using TRIzol reagent. RNA integrity and quality were assessed with a 5200 Fragment Analyzer employing the DNF-471-33-SS Total RNA 15 nt kit (Agilent Technologies, Santa Clara, CA, USA). Library construction and sequencing procedures followed previously published protocols.^27^ Briefly, 500 ng of total RNA served as input for poly(A) + mRNA enrichment using oligo-dT magnetic beads, and strand-specific libraries were generated with the TruSeq Stranded mRNA Library Preparation Kit (Illumina, San Diego, CA, USA) according to the manufacturer's recommendations (half-volume reaction). Libraries were amplified by 13 PCR cycles. Fragment size distribution of the indexed libraries (~290 bp) was verified by electrophoretic analysis on a 2100 Bioanalyzer using the DNA 1000 kit.^28^

Pooled libraries were supplemented with 1% PhiX control and sequenced in single-end mode (100 nt read length) on a NextSeq 2000 system (Illumina), targeting approximately 25 million reads per sample. FASTQ files were obtained after demultiplexing with bcl-convert (v4.2.4, Illumina).

As described before,^29^ raw Illumina reads underwent adapter removal and quality trimming using Cutadapt (v2.5;^30^ in NextSeq mode with a Phred score threshold of 20. Reads lacking remaining bases after trimming were excluded (parameters: –nextseq-trim = 20-m 1-a AGATCGGAAGAGCACACGTCTGAACTCCAGTCAC). Filtered reads were aligned to the rat reference genome (NCBI RefSeq assembly GCF_036323735.1/GRCr8) using STAR (v2.7.2b;^31^ with default settings and transcript annotations from RefSeq release RS_2024_02 for GRCr8. Gene-level exon counts were generated with featureCounts (v1.6.4, Subread package;^32^ based on the same annotation. Strand-specific counting was performed in reverse mode, including multi-mapping and multi-overlapping reads with fractional assignment (parameters: -s 2 -t exon -M -O –fraction). The resulting count matrix was analyzed for differential gene expression using DESeq2 (v1.24.0,^33^. Size-factor normalization was applied, and log2 fold-change shrinkage was performed with the betaPrior parameter set to TRUE. Genes were considered differentially expressed if the Benjamini–Hochberg adjusted *p*-value (padj) was <0.05 and the absolute log2 fold change was ≥1. Functional enrichment analysis of KEGG pathways was conducted with clusterProfiler (v4.8.1;^34^ was used to perform functional enrichment analyses based on KEGG pathways. Gene set enrichment analysis (GSEA) was performed using the GSEA function, incorporating DESeq2-derived log2 fold-change values for all evaluated genes.

## 16s-rRNA marker gene analysis

Genomic DNA was isolated from 200 mg of colonic content using the QIAamp PowerFecal Pro DNA Kit (Qiagen, Hilden, Germany) following the supplier's protocol. DNA yield and purity were determined with a NanoDrop spectrophotometer (Thermo Fisher).

Sequencing libraries were generated from 10 ng of extracted DNA applying a custom protocol based on Pichler et al. ^35^, as previously described.^27^ ^,^ ^36^ Libraries were amplified in 17 PCR cycles, and fragment size distribution of the indexed products (~397 bp) was evaluated using a 5200 Fragment Analyzer with the DNF-474-33 HS NGS Fragment 1–6000 bp kit.

Equimolar pooled libraries were supplemented with 10% PhiX control and sequenced on an Illumina MiniSeq instrument in paired-end mode (2 × 150 nt), aiming for approximately 600,000 reads per sample. Custom sequencing primers were added to the standard Illumina primer mix for both read and index reactions. Demultiplexed FASTQ files were produced using bcl-convert (v4.0.3, Illumina). Sequence processing was performed with the QIIME2 framework (version 2023.9.2;^37^. Paired-end reads were subjected to quality control, trimming, and merging using the DADA2 plugin, resulting in high-confidence amplicon sequence variants (ASVs), with chimera filtering incorporated in this workflow.^38^

Alpha diversity metrics, including Faith's Phylogenetic Diversity,^39^ and Pielou's evenness,^40^ were computed using q2-diversity after rarefying the feature table to a sampling depth of 430,000 sequences per sample. Beta diversity was assessed using Bray–Curtis dissimilarity, ^41^ and visualized using Principal Coordinates Analysis (PCoA).

Taxonomy was assigned to ASVs as carried out using the q2-feature-classifier plugin,^42^ with a naïve Bayes classifier trained on region-specific reference sequences derived from the Greengenes2 16S rRNA database (v2022.10,^43^, matching the amplified regions analyzed in this study. Differential abundance testing was performed in R (v4.3.2,^44^ using ANCOM-BC2 (v2.4.0,^45^ to detect genera with significant differences between groups. Correlation Network analysis was conducted with the NetCoMi package (v1.1.0,^46^ applying Spearman correlation, centered log-ratio transformation for normalization of compositional data, and multiplicative simple replacement to handle zero values.

### Intestinal contents preparation for cell culture experiments

Colonic contents were prepared, as described previously.^25^ Frozen colonic content (stool) was resuspended in distilled water to yield a final concentration of 100 mg/mL. Samples were homogenized for 10 min using a TissueLyzer (Qiagen). The homogenates were then centrifuged at 13,000 rpm for 10 min at 4 °C. The supernatants were passed through a 70 μm nylon cell strainer (Falcon), combined separately for LFD and HFD groups, aliquoted, and preserved at −80 °C until further use. The 70 μm filtration step preserves the biological activity of colonic-content-derived factors and represents the complex colonic-content-derived fraction to reflect the physiological complexity of the intestinal lumen.

### Epithelial regeneration (wound healing) assay

2D organoid monolayers were cultured as described before and epithelial wound healing assays were carried out as described previously.^17^ ^,^ ^18^ Briefly, organoids were harvested, washed, and mechanically dissociated to obtain a single-cell suspension. For plating, cells were seeded into 12-well TPP® tissue culture plates (TPP, Trasadingen, Switzerland) pre-coated with 10% Geltrex LDEV-Free Reduced Growth Factor Basement Membrane Matrix (Gibco). Experiments with murine intestinal organoids were initiated once cultures had reached confluence, as previously described.

For two-dimensional assays, organoid-derived monolayers were maintained in either 6-well or 8-well plates (Ibidi, Gräfelfing, Munich, Germany). Upon reaching confluence, a standardized scratch was introduced using a 10 μL pipette tip. All wound inflictions were carried out by the same investigator under blinded conditions, followed by replacement of the culture medium.

Microscopic images were captured at 0, 24 and 48 h after scratching. Wound closure was quantified with ImageJ software by comparing the remaining wound area at each time point to the initial wound area measured immediately after scratching (*t* = 0). An *n* value for the wound healing experiments is one biological replicate which is carried out in duplicates or triplicates. The mean between the duplicates represents one *n* and is presented as one dot in the shown in the bar plots of the figure.

Monolayers were incubated with medium supplemented with 10% HFD or LFD colonic content, either alone or in combination with pharmacological inhibitors: MRS 2578 (N,N″-1,4-butanediylbis[N′-(3-isothiocyanatophenyl)thiourea]; Merck/Sigma-Aldrich, Darmstadt, Germany) at 1 μM; FR180204 (Biozol, Hamburg, Germany) at 1 μM; or FR180204 (MedChemExpress, New Jersey, USA) at 5 μM.

Cell proliferation was assessed by Ki67 immunofluorescence staining. Images were acquired using identical microscope settings for all experimental groups by a blinded observer. Ki67-positive nuclei were quantified using ImageJ (NIH) by determining the number of Ki67-positive nuclei relative to the total number of DAPI-positive nuclei within each high-power field (HPF). Four HPFs were analyzed per experiment, and the mean value was used to represent one biological replicate (*n* = 1). Data are expressed as the percentage of Ki67-positive cells per HPF.

### TEER measurements

Organoids were dissociated into single cells and seeded onto 24-well filters coated with a mixture of medium and Geltrex (Thermo Fisher 1:10). 300 μl media was added to the apical side, and 1 ml to the basolateral side of the filter. For content experiments 10% of either HFD or LFD content were added to the apical side. For UDP-glucose experiments 100 μmol/l UDP-glucose was added to the apical side. Filters were inserted into an 8-well Trans-Filter-Array (Applied Biophysics, Troy, NY, USA) and electrical resistance was measured at 400 Hz for 48 h using a ECIS Zθ (Applied Biophysics).

### Untargeted metabolomics

Untargeted metabolomic analysis was performed as previously described.^47^ This approach was applied as an unbiased, exploratory strategy to broadly assess metabolic differences between groups prior to targeted analyses. Analysis was performed using Acquity Ultra Performance LC coupled to a Synapt G2 HDMS equipped with an electrospray ionization source (Waters, Milford, MA, USA). Extracted metabolites in 5 μl injected samples were separated on a BEH C18 column (2.1 × 100 mm, 1.7 μm, Waters) using a linear binary solvent gradient of 0%–50% eluent B over 10 min at a flow rate of 0.3 mL/min and a temperature of 40 °C. After the gradient, the column was washed with 100% eluent B for 3 min and then equilibrated with 0% eluent B for 2 min. Eluent A was 0.1% formic acid (FA) in water and eluent B was acetonitrile (ACN). The MS was operated in positive and negative electrospray ionization mode with a capillary voltage of 1.5 kV. The source temperature was set at 120 °C and nitrogen was used as the desolvation gas (at 350 °C, flow = 800 L/h). Centroid data were acquired with a mass range of 50–1200 Da using leucine-enkephalin for single point internal calibration in MS mode. MassLynx 4.1 was used to operate the instrument. After the measurements, the raw data were pre-processed using Progenesis QI (v1.0) and the pre-processed data were further analyzed using Metaboanalyst 6.0. All metabolomic analyses were performed on a total of seven samples (HFD *n* = 3; LFD *n* = 4).

### Targeted metabolomics

Targeted metabolomic analysis was performed as previously described.^48^ This approach was used to enable accurate identification and quantification of predefined metabolites following the exploratory untargeted analysis. Water-soluble metabolites were extracted by adding 500 μL of ice-cold methanol/water (80:20, v/v) containing internal standards (0.01 μM lamivudine and 1 μM each of D2-glucose, D4-succinate, D5-glycine, and 15N-glutamate; Sigma-Aldrich). Following centrifugation, supernatants were evaporated to dryness using a rotary evaporator (Savant, Thermo Fisher). The dried extracts were reconstituted in 75 μL ACN/H₂O/formic acid (5/94.9/0.1, v/v/v) and applied to a pre-activated C18 solid-phase extraction column (Strata C18-E, 50 mg/1 mL; Phenomenex, Aschaffenburg, Germany) that had been conditioned with 0.5 mL ACN and 0.5 mL of the reconstitution solvent. Unbound metabolites were removed with 75 μL of the same solvent, and bound metabolites were sequentially eluted with 150 μL ACN/H₂O/FA (50/49.9/0.1, v/v/v) followed by 225 μL ACN/H₂O/FA (90/9.9/0.1, v/v/v). Each fraction was collected separately and evaporated to dryness.

Residues of unbound metabolites were reconstituted in 75 μL of ACN/H₂O (50:50, v/v) containing 5 mM ammonium acetate (NH4OAc), whereas eluted fractions were dissolved in 175 μL of the same solution. Metabolite analysis was conducted by LC-MS, with 3 μL of each sample injected onto an XBridge Premier BEH Amide column (2.5 μm, 100 × 2.1 mm; Waters). Eluent A consisted of 5 mM NH4OAc in ACN/H₂O (40/60, v/v) and eluent B of 5 mM NH4OAc in ACN/H₂O (95/5, v/v), delivered at 200 μL/min and 45 °C using a DIONEX Ultimate 3000 UHPLC system (Thermo Fisher). A linear gradient was applied, increasing from 0% to 90% eluent A over 23 min (after 2 min initial hold), followed by a 16-min wash at 90% eluent A and a 7-min re-equilibration at 0% eluent A.

The remaining extract was derivatized for polar carboxylic metabolites by dissolving in 20 μL of O-benzylhydroxylamine and N-(3-dimethylaminopropyl)-N′-ethylcarbodiimide (0.5 M) in pyridine buffer (86 mL H₂O, 10.8 mL 6 M HCl, 8.6 mL pyridine). After 1 h at room temperature, derivatized metabolites were extracted with 0.3 mL ethyl acetate, washed with 50 μL H₂O, and dried under nitrogen at 45 °C. Residues were reconstituted in 75 μL 5 mM NH4OAc in ACN/H₂O (50:50, v/v) and centrifuged. Three μL were injected onto an Atlantis Premier BEH C18 AX column (2.5 μm, 100 × 2.1 mm; Waters) for LC separation. Eluent A consisted of 0.1% formic acid in ACN/H₂O (5/95, v/v) and eluent B of 0.1% formic acid in ACN/H₂O (90/10, v/v), delivered at 200 μL/min at 45 °C. A linear gradient was applied, beginning at 10% B after a 2-min hold, ramping to 100% B over 20 min, followed by a 10-min wash at 100% B and 5-min equilibration at 10% B.

High-resolution MS analyses were performed on a Q Exactive mass spectrometer with HESI probe (Thermo Fisher Scientific) in alternating positive/negative full-scan mode (m/z 69–1000, 70 K resolution) using the following ESI parameters: sheath gas 30, auxiliary gas 1, sweep gas 0, aux gas heater 120 °C, spray voltage 3 kV, capillary 320 °C, S-lens RF 50. Data were processed with TraceFinder™ V5.1 (Thermo Fisher Scientific, Bremen, Germany), integrating peaks from extracted ion chromatograms of monoisotopic metabolite masses (±3 mMU) for quantification.

### Statistics

Data are presented as mean ± S.E.M. unless otherwise stated. All statistical tests were performed on GraphPad Prism 10.0 software. Data normality was assessed using QQ-plots to visually inspect the distribution of the data. For comparisons between two independent groups, an unpaired *t*-test was applied, while for paired data, a paired *t*-test was used. The relationship between variables was assessed using Spearman's rank correlation coefficient. For comparisons involving more than two groups, one-way or two-way ANOVA was applied as appropriate, followed by multiple-comparison testing. For non-normally distributed data, the Mann–Whitney U test was used. Permutational analysis of variance (PERMANOVA) was employed to assess the effect of group differences based on Bray–Curtis dissimilarities. PERMANOVA was performed using 9999 permutations in R 4.3.3. For all analyses, a *p*-value < 0.05 was considered statistically significant.

## Results

### Rats on a HFD developed an obese phenotype with impaired glucose tolerance and loss of intestinal barrier function

We first phenotyped a cohort of adult male Wistar rats maintained on a high-fat diet for 8 weeks (56 d) in comparison to a cohort that received low-fat diet. HFD rats gained considerably more weight than their LFD counterparts and had significantly higher body weights at the time of sacrifice after 56 d (575 ± 8 g vs. 513 ± 10 g, *p* < 0.001, Figure 1A). This magnitude of weight gain is consistent with previously reported effects of high-fat diets in adult male Wistar rats, confirming the development of an obese phenotype.^49^ At this time point, HFD rats showed increased serum LPS levels in the portal vein compared with LFD rats, indicating impaired intestinal barrier function (Figure 1B, *p* < 0.05). Due to interindividual variability of the LPS levels, correlation analyses were carried out which supported the correlation between increased serum LPS levels and body weight (r = 0.7667, *p* < 0.01). Additionally, HFD rats exhibited impaired glucose tolerance, illustrated by elevated fasting blood glucose levels (*p* < 0.01, Figure 1C) and an increased area under the curve of an oral glucose tolerance test (OGTT) (*p* < 0.001, Figure 1D).

**Figure 1.**
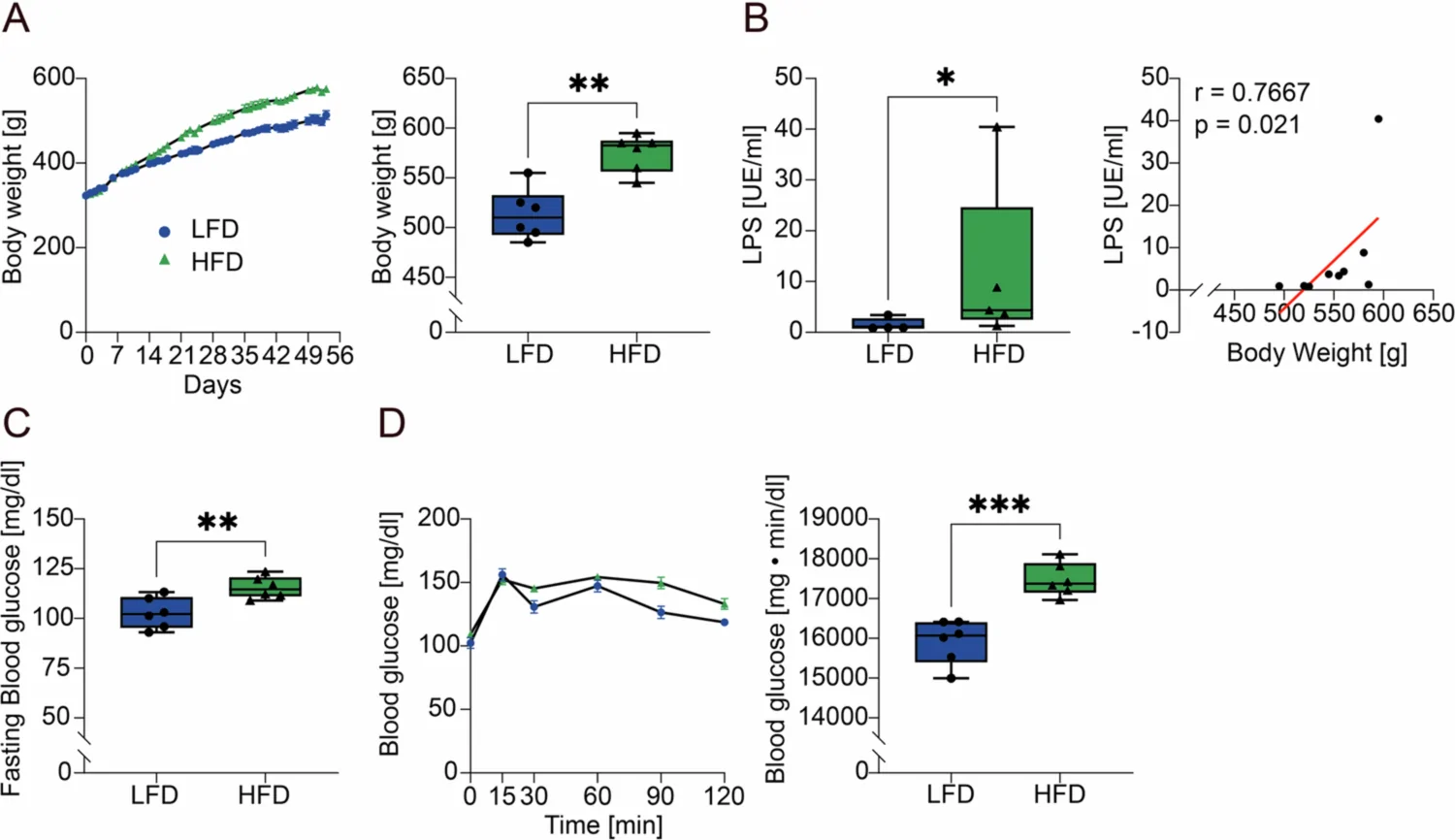
HFD induces obesity, impaired intestinal barrier function and impaired glucose homeostasis. (A) Body weight over time per group in rats on low-fat diet (LFD) or high-fat diet (HFD). Boxplots show the final body weights at the time of sacrifice for the individual rats (*n* = 6). (B) LPS levels measured in portal vein serum and correlation of serum LPS levels with final body weights at the time of sacrifice as determined by Spearman correlation (*n* = 4–5). Hemolytic samples were excluded from the measurements, no outliers were excluded from the analysis. (C) Fasting blood glucose levels after an eight hour fasting period (*n* = 6). (D) Blood glucose levels over time per group after an oral glucose challenge. Boxplots show the corresponding area under the curve (AUC) for individual rats (*n* = 6). Data are presented as mean ± SEM. *p < 0.05, **p < 0.01, ***p < 0.001, as determined by unpaired Mann-Whitney test. The solid regression line indicates least squares fit of data.

Next, we assessed microbiota composition of the rats on a HFD in comparison to the LFD cohort. Rats on a HFD exhibited significantly altered microbial community composition, both in beta-diversity, as assessed by PERMANOVA analysis of the Bray–Curtis-Index (*p* < 0.004, Figure 2C) and richness (*p* < 0.006, Figure 2A), while evenness remained unchanged (Figure 2B). In line with previous findings,^50^ obese rats tended to have a higher Firmicutes/Bacteroidota ratio compared to LFD rats (*p* = 0.052, Figure 2D) demonstrating the comparability of our models with previous models in the literature. In Figure 2C, each dot represents the microbial community of an individual rat, and distances between dots reflect Bray–Curtis dissimilarity. Ellipses indicate the 95% confidence interval around each group's centroid, highlighting the clustering of samples within groups. Differential abundance analysis with ANCOMBC2 revealed that several genera were decreased, with *Prevotella* being the most prominent, while *Pseudobutyricicoccus* was the most increased genus compared to the LFD group (Figure 2E). The differential microbial network analysis revealed distinct correlation patterns between microbial genera under LFD and HFD conditions (Figure 2F). Specifically, the associations among certain genera shifted significantly between the dietary interventions. In the association network, differentially correlated genera included *Bifidobacterium*, *Akkermansia*, *Alloprevotella*, and *Limosilactobacillus*, which exhibited altered interactions between LFD and HFD. Under LFD, *Bifidobacterium* showed a strong positive correlation with *Butyribacter* and *14-2*, whereas these associations were diminished or altered under HFD. Similarly, *Akkermansia* displays a negative correlation with *Alloprevotella* under LFD, while this relationship shifted towards a neutral or weak correlation in HFD conditions (Figure 2G).

**Figure 2.**
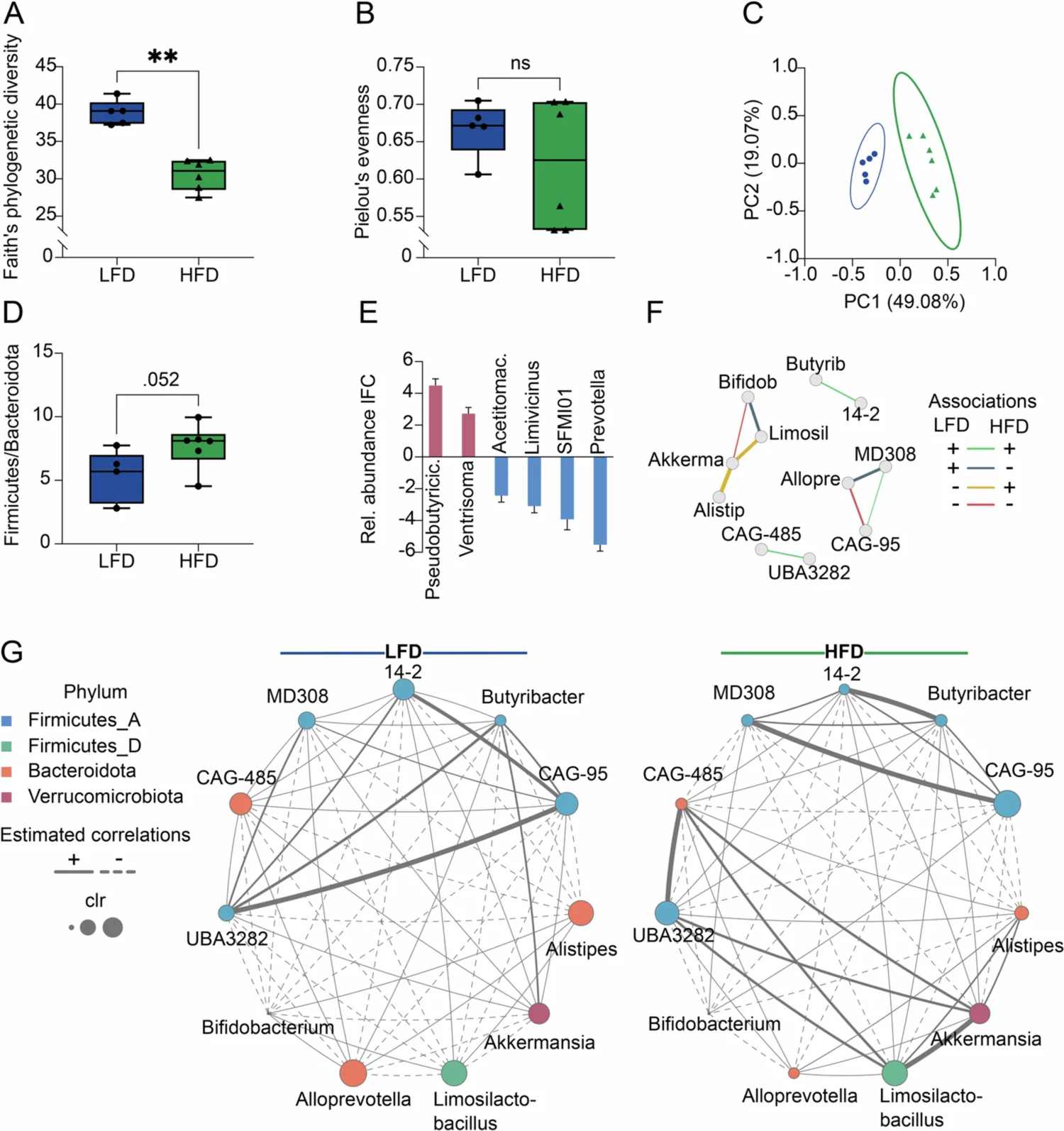
HFD changes intestinal microbial community composition in the colon. (A and B) Alpha diversity measurements of community composition with (A) Faith's phylogenetic diversity as a measure of richness and (B) Pielou's evenness as a measure of community evenness. (C) Beta diversity plot of Bray–Curtis dissimilarity index. (D) Ratio of Firmicutes' to Bacteroidota relative abundance. (E) Differential abundant genera relative to LFD group. (F) Differential network plot of genera differentially correlated between LFD and HFD groups. (G) Association network of differentially correlated genera, showing associations between genera for LFD (left) and HFD (right) groups. For all groups, *n* = 5–6; due to poor *16s-rRNA* quality one animal was excluded from the analysis. **p < 0.01, determined by unpaired Mann-Whitney test. Correlation analysis was performed using Spearman correlation.

Taken together, these findings support the conclusion that the animal model used here closely matches the clinical features of obesity and metabolic syndrome including impaired glucose homeostasis, impaired intestinal barrier function and altered microbial community composition.

### Obese rats display local intestinal inflammation which is accompanied by changes of junctional proteins in the colon

Since chronic low-grade inflammation has been linked to the metabolic changes in obesity, we next assessed the extent of systemic inflammation in our model. Systemic and portal vein blood samples taken from rats subjected to either LFD or HFD after 8 weeks were analyzed for a potential increase of different pro-inflammatory cytokines using multicytokine assays. Both LFD and HFD groups showed no differences neither in peripheral blood nor in portal vein (Figure S1). In contrast, analysis of colonic tissue revealed pronounced local inflammatory changes in HFD fed rats. First, in western blots we found increased levels of TNFα in the colon of HFD rats compared with LFD rats (*p* = 0.008, Figure 3A). Based on our hypothesis that innate immune activation contributes to this phenotype, we assessed the mRNA expression of the toll-like receptors (Tlr) 2 and 4 by qRT-PCR. Both receptors showed increased expression in the colon (*p* = 0.029; Figure 3B). Thereafter, bulk transcriptome sequencing of colon lysates was carried out which revealed a clustering between LFD and HFD rats. GSEA revealed a strong activation of the pro-inflammatory MAPK-pathway (NES = 1.6, *q* < 0.001, Figure 3C). In addition, pathways related to PI3K-Akt signaling (NES = 1.5, *q* < 0.001), cell cycle (NES = −2.1, *q* < 0.001) and cytoskeleton (NES = 2.4, *q* < 0.001) were enriched in HFD colon lysates. Of note, TLR2/4 activation is known to engage all of these pathways with a substantial overlap^51^ which may explain why the latter upstream regulators were not individually enriched in GSEA.^52^ Nonetheless, all these findings together pointed to a local inflammatory phenotype in the gut following HFD feeding.

**Figure 3.**
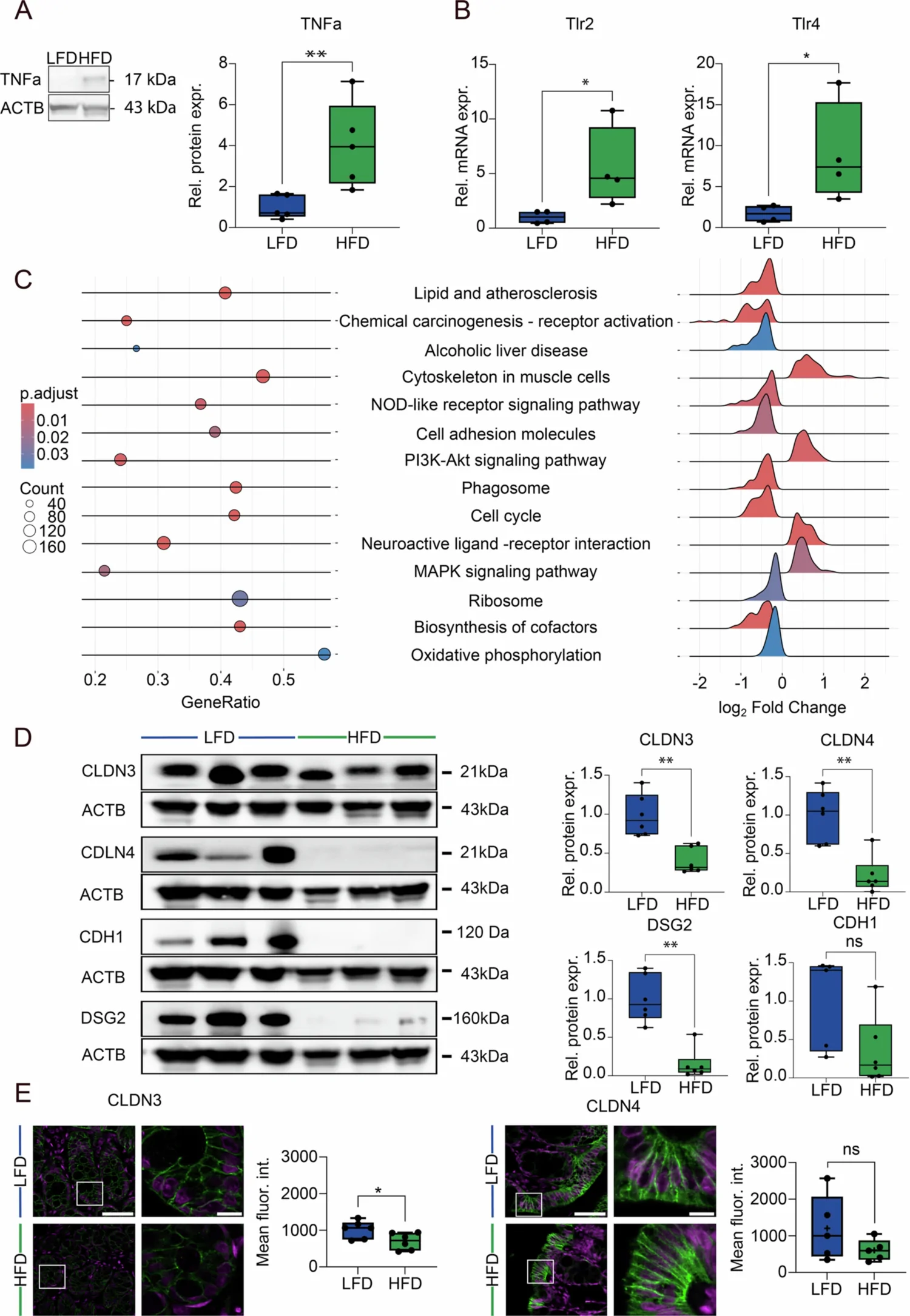
HFD induced an inflammatory phenotype and barrier impairment in the colon of rats. (A) Representative Western blot bands of TNFα in colon sections and corresponding quantification (*n* = 5). (B) Expression of Tlr2 and 4 mRNA in the colon (*n* = 4). (C) Dotplot and corresponding ridge plot for enriched pathways as determined by KEGG gene set enrichment analysis (*n* = 4). (D) Representative Western blot bands of intestinal barrier proteins and corresponding quantification. (E) Representative immunofluorescence images of tight junction proteins CLDN3 and CLDN4 and corresponding quantification of fluorescence intensity (*n* = 5). *p < 0.05, **p < 0.01 as determined by unpaired Mann-Whitney test; scale bar is: 50 μm.

To assess whether this inflammatory state was associated with changes of IEB composition, we analyzed the expression of desmosomal (Desmoglein2 (DSG2)), tight junction (Claudin-3 (CLDN3), Claudin-4 (CLDN4)) and adherens junction (E-Cadherin (CDH1)) proteins (Figure 3D). Western blot analysis revealed decreased levels of DSG2 (*p* < 0.01) and of tight junction proteins CLDN3 and CLDN4 (*p* < 0.01, respectively) in HFD rats. These findings were substantiated by immunofluorescence staining of colonic sections, which confirmed overall reduction of CLDN3 in the HFD group (*p* < 0.05, Figure 3E).

### Colonic contents from HFD rats cause impaired intestinal epithelial regeneration

Changes in Tlr2 and 4 expression, locally increased TNFα levels associated with activation of pathways involved in intestinal inflammation led us to hypothesize that alterations in the colonic microbial community composition contribute to impaired IEB function in obesity. To test this, we investigated the effects of colonic content-derived solutions from HFD and LFD rats^25^ in Caco-2 and murine organoid models. Caco-2 cells exposed to HFD-derived content showed a significant decrease in measurements of transepithelial-electrical resistance (TEER) after 48 h compared with LFD content (*p* = 0.0002, Figure 4A). Thereafter, colonic contents of each model were incubated with 2D organoid monolayers followed by (TEER) measurements. Interestingly, 2D organoids which were grown to confluency when measurements had started, were unaffected by HFD-derived colonic content (Figure 4B). However, when HFD-derived content was applied to proliferating, non-confluent organoids, TEER failed to increase compared to monolayers subjected to LFD-derived content, suggesting that HFD-derived luminal factors impair epithelial maturation or epithelial growth (*p* = 0.001, Figure 4C). To further substantiate this observation, 2D organoid monolayer scratch wound assays were performed as a functional read-out of epithelial regenerative capacity which includes both cell proliferation and migration. Both LFD and HFD contents affected epithelial regeneration compared with untreated controls, with the strongest effect following exposure to HFD content (*p* < 0.01) (Figure 4D and E).

**Figure 4.**
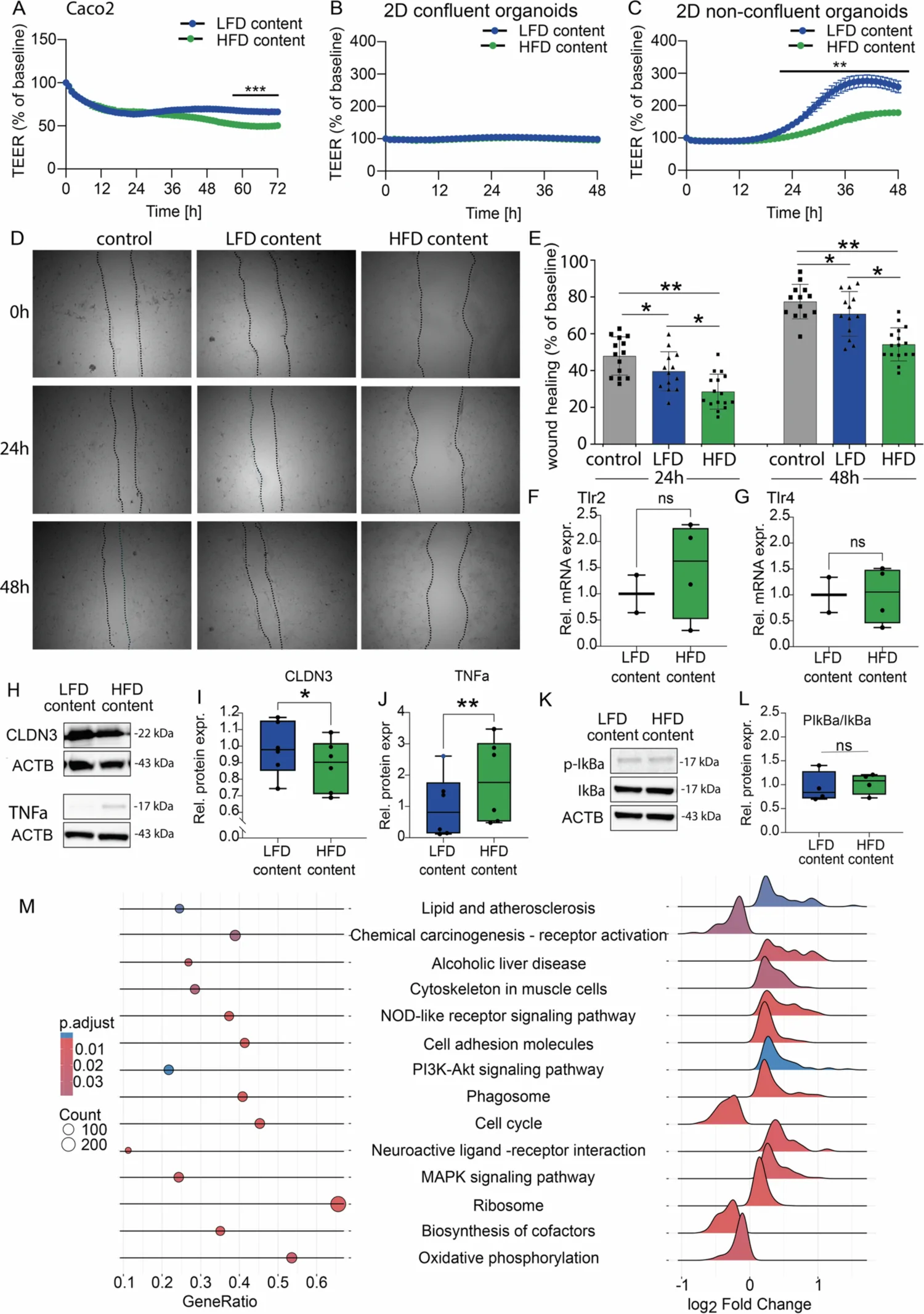
(A–C) TEER of Caco-2 cells (*n* = 12) (A), 2D confluent organoid monolayers (*n* = 8) (B) and 2D non confluent monolayers (*n* = 9) (C) treated with colonic contents from LFD and HFD rats. (D) Representative Images of wound healing assays in 2D organoid monolayers of murine organoids under the different conditions. (E) Quantification of wound healing expressed as area of scratch relative to control (*n* = 14–16). (F–G) Expression of Tlr2 (F) and 4 (FG mRNA in murine organoids (*n* = 3). (H) Representative Western blot bands of CLDN3 and TNFα in murine organoids and corresponding quantification (*n* = 6). (K–L) Representative Western blot bands of phosphorylated and total IkBa in murine organoids (K) and corresponding quantification (L). (M) Dotplot and corresponding ridge plot for enriched pathways in murine organoids as determined by KEGG gene set enrichment analysis (*n* = 3).

While Tlr2 and Tlr4 mRNA expression remained unchanged in the organoid model (Figure 4F and G), further analysis of the effects of colonic content on intestinal barrier composition and inflammatory signaling revealed a phenotype comparable to that observed in vivo: Western blotting showed decreased CLDN3 levels (*p* = 0.028), while epithelial TNFα levels were increased (*p* = 0.007, Figure 4H–J) in organoids treated with HFD-derived content. However, the ratio of phosphorylated IkBa to total IkBa was not altered in organoids exposed to HFD-derived content (Figure 4K and L). Consistent with the findings in rat colons, KEGG pathway analysis revealed enrichment of the MAPK pathway in organoids treated with HFD-derived content (NES = 1.4, *q* < 0.0021, Figure 4M).

### Metabolomic profiles of colonic contents differ significantly between HFD and LFD groups with increased UMP/UDP-related metabolites in the colonic content of HFD rats

In order to explore possible mechanisms behind the observed effects, we subjected the colon contents used in the cell culture experiments to LC-MS based metabolite analysis. Untargeted metabolomics was initially performed as an exploratory approach to capture global metabolic differences between groups. Untargeted metabolomics of 809 aligned features revealed distinct profiles between the two groups: the first principal component accounted for 72.3% of the total variation and clearly separated the LFD and HFD groups (Figure 5A), a result confirmed by hierarchical clustering analysis (Figure 5B). Next, univariate statistical analysis was performed to identify significant differences in metabolite levels between the LFD and HFD. Notably, 40% of the annotated metabolite features (330) differed significantly (*p* < 0.05, fold change > 2), with 47 and 56 detected exclusively in the LFD and HFD, respectively (Figure 5C). We then manually selected the top ten metabolite features but were unable to identify them by MS/MS product ion scans or by searching common metabolomics databases, including mzCloud, MassBank, and the Human Metabolome Database. Thus, despite revealing clear group separation, the untargeted approach did not yield sufficiently annotated metabolites for biological interpretation. Our results indicate that the metabolite profile of the HFD differs markedly from that of the LFD, although further studies are needed to elucidate structures of the metabolites specific to LFD and HFD. As a second approach, we performed targeted metabolomics on 206 detectable hydrophilic primary metabolites; hierarchical clustering revealed clear separation between the HFD and LFD groups (Figure 5D), confirming the untargeted metabolomics results (Figure 5A and B). In contrast to the untargeted analysis, this approach enabled reliable identification and quantification of specific metabolites. A key finding of the targeted analysis was the higher levels Uridine monophosphate (UMP), uridine diphosphate (UDP), uridine diphosphate N-acetylglucosamine (UDP-GlcNAc), and uridine diphosphate glucose (UDP-Glc) in the HFD group compared with the LFD group (Figure 5E).

**Figure 5.**
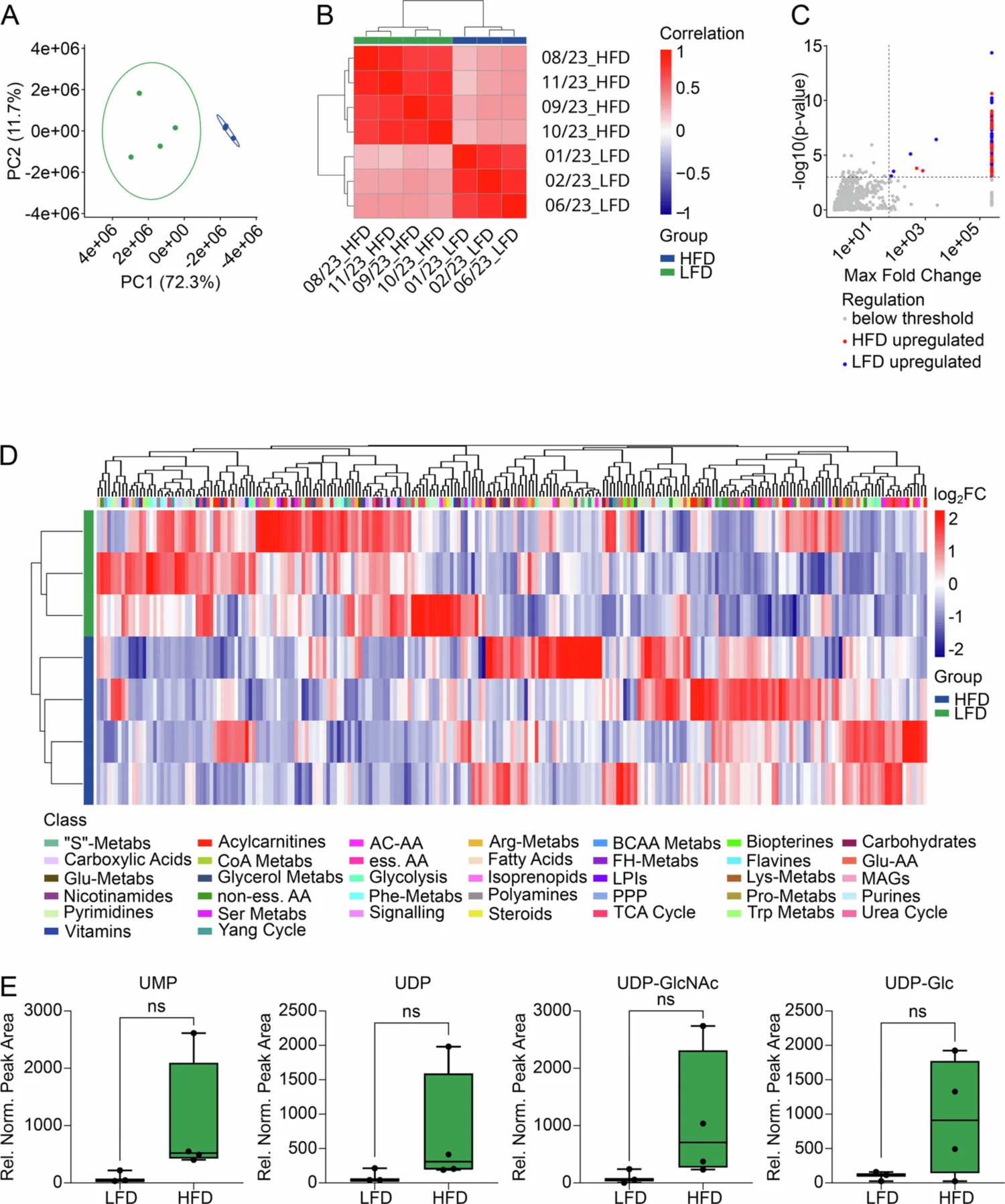
HFD alters global colonic metabolite profiles. (A) principal coordinates analysis (PCoA) of colonic content composition, each sample is represented by a point. Ellipses show confidence regions for each group. (B) Heatmap showing clustering of samples based on their overall similarity. (C) Dot-plot of most upregulated metabolite features in HFD and LFD samples, cut-off values are *p* < 0.001 and fold-change > 1000. (D) Hierarchical clustering heatmap of metabolites included in the targeted analysis. Columns show compounds, with a color key indicating the compound class. Data are scaled by column to normalize expression levels. The color scale indicates expression (red = high, blue = low). Rows show samples, using hierarchical clustering. LFD and HFD groups are denoted by a color key. (E) Relative normalized peak Area (Rel. Norm. Peak Area) of UDP and related compounds relative to LFD. Boxplots show the median (horizontal line), with whiskers indicating minimum and maximum values, *n* = 3–4. ns = no difference, as determined by unpaired *t*-test.

### Impaired HFD-induced epithelial regeneration is mediated by UDP-induced P2Y_6_-receptor activation

Since free UDP and UDP-glucose have previously been implicated in intestinal inflammation, we next sought to determine whether either metabolite affects intestinal epithelial integrity and regeneration under conditions of obesity.^53^ ^,^ ^54^ UDP-glucose alone had no effects on intestinal wound healing, TEER, and MAPK-signaling as assessed by Western blot analysis (Supplementary Figure S2). Therefore, we hypothesized that free UDP might be causative for the observed effects. Indeed, application of UDP significantly impaired epithelial wound healing in 2D organoid monolayer scratch wound assays compared with controls or scratch wound assays exposed to LFD content (Figure 6A and B; *p* < 0.05). As UDP signaling is known to be mediated via P2Y_6_-receptor, we applied the specific receptor antagonist MRS2578. The treatment with this antagonist attenuated the UDP-mediated impairment of epithelial wound healing (Supplementary Figure S2), indicating that the effects of UDP on epithelial regeneration are P2Y_6_-dependent. Next, we investigated whether blockade of P2Y_6_-receptor with MRS2578 modulates HFD-induced impairment of epithelial wound healing. Application of MRS2578 blunted HFD-induced loss of epithelial wound healing (Figure 7A and B). MAPK-signaling is known to occur downstream of P2Y_6_-receptor activation and was found to be increased both *in vitro* and i*n vivo* under HFD condition (Figures 3C and 4M). Therefore, we next examined whether inhibition of MAPK signaling could attenuate HFD-induced defects in intestinal epithelial regeneration. Indeed, while treatment with the ERK inhibitor FR180204 resulted in an overall reduction in wound healing in organoids exposed to LFD content, compromised wound healing under HFD condition was significantly improved following application of ERK inhibitor (Supplementary Figure S3). Given the involvement of ERK/MAPK signaling downstream of P2Y_6_ we tested whether proliferation is impaired following HFD application. Therefore, we used Ki67 staining and estimated the number of ki67-positive cells following wound healing after exposure of cells LFD and HFD incubation. Consistent to the observations on wound healing, exposure to LFD content did not change the number of ki67-positive cells compared to untreated organoid cultures (67% ± 4% vs. 62% ± 4%). However, co-incubation of organoids with HFD content resulted in a reduction of ki67-positive cells to 41% ± 5%. Loss of ki67-positive cells after exposure to HFD was blunted by co-incubation with MRS2578 (62% ± 6%) while neither MRS2578 (63% ± 5%) alone nor neither together with LFD content (64% ± 5%) affected the number of ki67-positive cells (Figure 7C and D).

**Figure 6.**
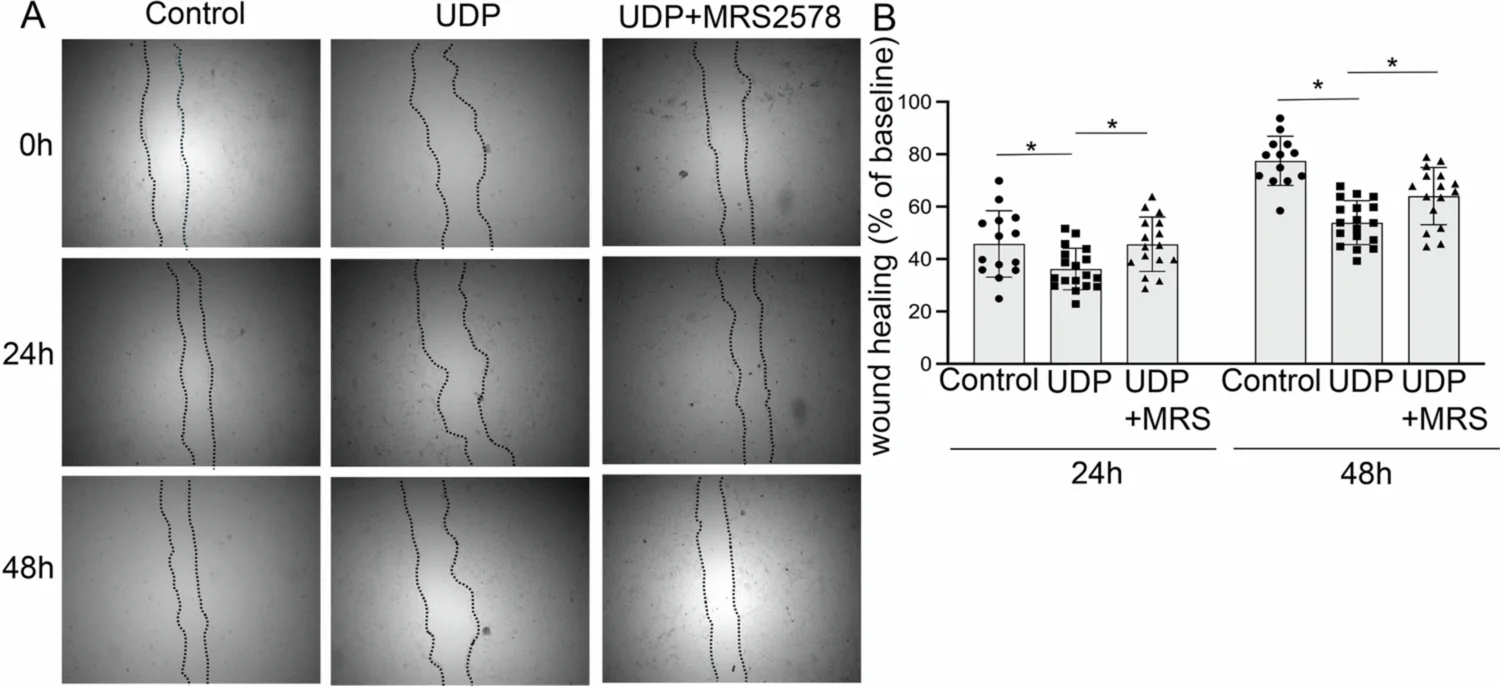
(A) Representative Images of wound healing assays in 2D monolayers of murine organoids treated with UDP and MRS2578. (B) Quantification of wound healing expressed as Area of scratch relative to control after 24 h and after 48 h (*n* = 16–18; *p < 0.05; ordinary two-way Anova).

**Figure 7.**
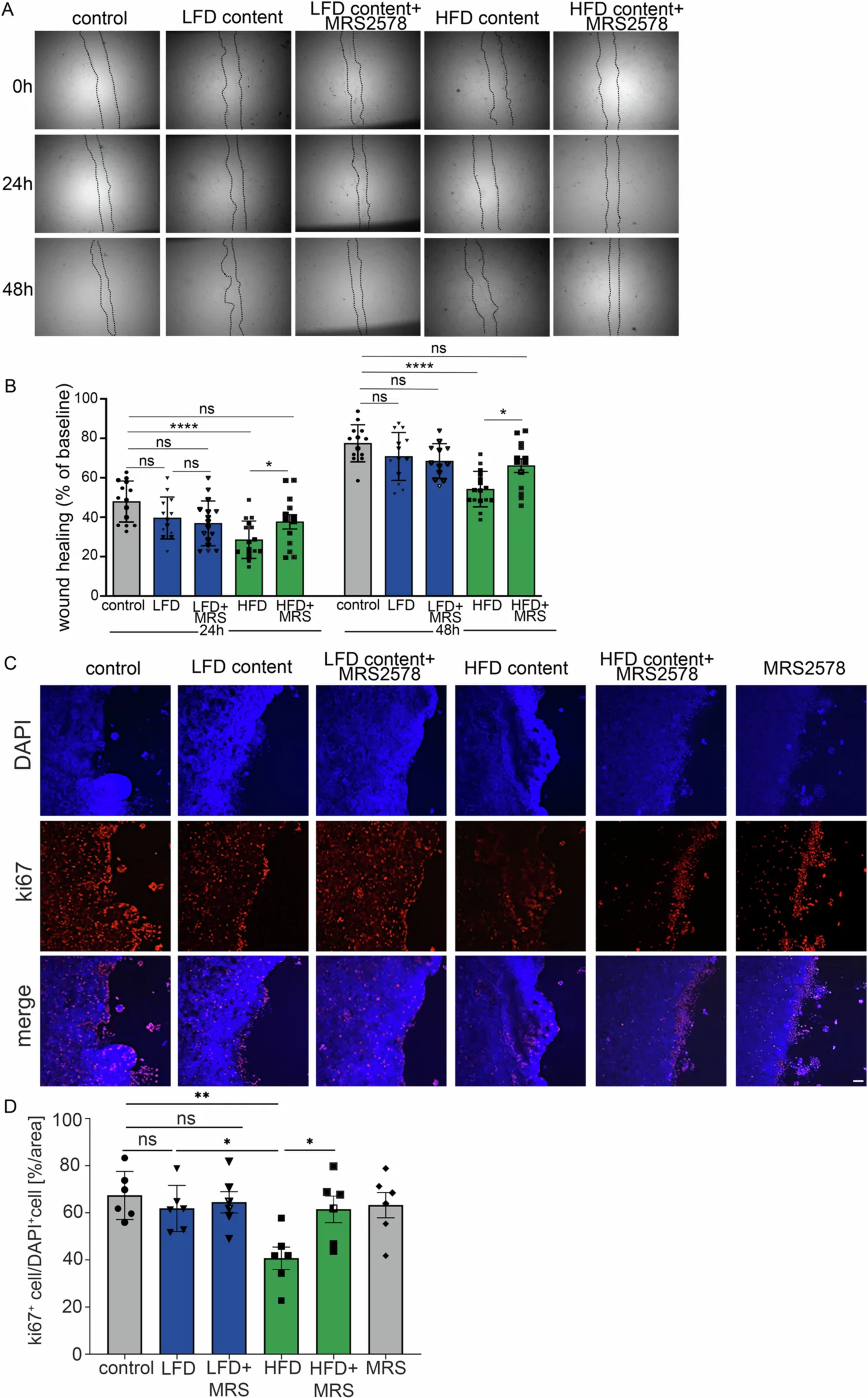
(A) Representative images of wound healing assays in 2D monolayers of murine organoids treated with LFD or HFD content and MRS2578. (B) Quantification of wound healing expressed as Area of scratch relative to control after 24 h and 48 h are shown (*n* = 14-16; *p < 0.05; ****p < 0.0001; two-way Anova). (C) Representative staining for DAPI or ki67 of 2D monolayers of murine organoids treated with LFD or HFD content and/or MRS2578 are shown after 48 h. (D) Quantification of the number of ki67-positive cells in relation to DAPI-positive nuclei/HPF were counted after 48 h (*n* = 6; *p < 0.05: **p < 0.01; ordinary one-way Anova).

Finally, growth kinetics of 3D organoids incubated with colonic content were continuously monitored using a live-cell imaging system over 72  h in presence of LFD or HFD contents (Figure 8A). Representative time-lapse imaging suggested that growth of organoids was impaired in the presence of HFD content (Supplementary video) and that this effect was attenuated in the presence of MRS2578 (Supplementary video). Unbiased quantitative analysis showed a growth rate of 0.95 ± 0.15 μm/h in the untreated control and 0.88 ± 0.32 μm/h following exposure of organoids to LFD content (Supplementary Figure S4, Figure 8B and C). Doubling time was 34.98 ± 3.46 h and 46.12 ± 10.22 h under the latter conditions. Administration of HFD contents to organoids was associated with a numerically lower growth rate of 0.58 ± 0.13 μm/h, corresponding to a 34% reduction of organoid growth to 0.58 ± 0.13 μm/h and a longer doubling time to 62.62 ± 9.06 h. Application of MRS2578 to the organoids alone did not markedly alter growth patterns (0.79 ± 0.30 μm/h) and doubling time (48.28 ± 15.71 h) (Supplementary Figure S4, Figure 8B and C). Application of MRS2578 to HFD resulted in a numerically higher growth rate to 0.97 ± 0.13 μm/h and shortened doubling time to 53.41 ± 11.86h while MRS2578 in combination with LFD led to increased growth to 1.4 ± 0.36 μm/h and to a shorter doubling time of 30.69 ± 7.81 h. As the unbiased analysis included both growing and non-growing organoids, considerable variability in individual organoid growth kinetics was observed. Consequently, although numerical differences were apparent between groups, these did not reach statistical significance. Nevertheless, the observed trends are consistent with the findings from the wound-healing assays and support a potential role of P2Y_6_ signaling in HFD content-associated impairment of epithelial regeneration.

**Figure 8.**
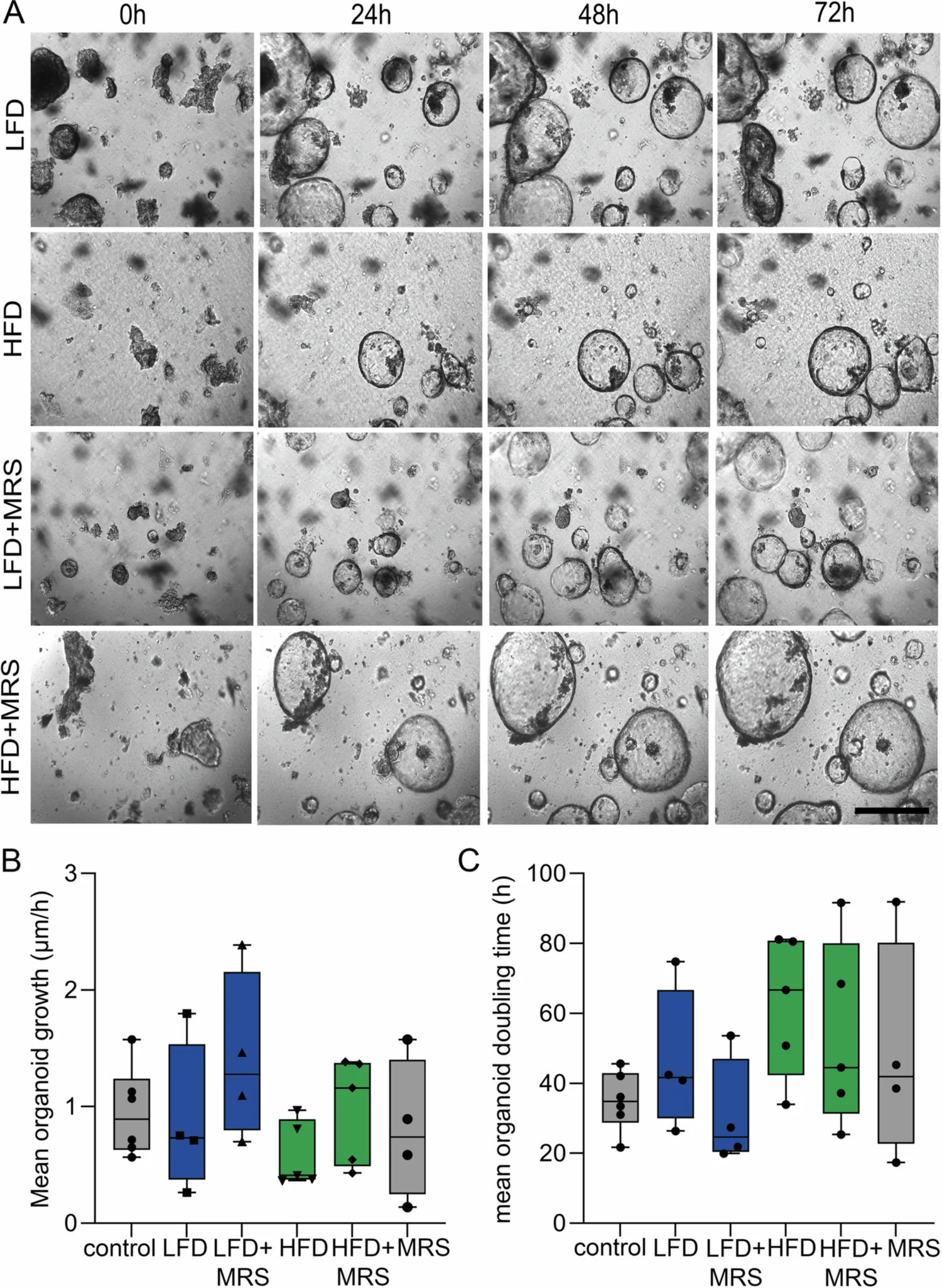
Representative micrographs of the same 3D organoid cultures are shown at the start of the experiment (0 h) and after 24, 48, and 72 h of incubation. Organoids were incubated with colonic contents from low-fat diet (LFD)- or high-fat diet (HFD)-fed animals in the absence or presence of the P2Y6 receptor antagonist MRS2578. Representative images of untreated control cultures and cultures treated with MRS2578 alone are provided in Supplementary Figure S5. Scale bar = 400 μm. (B) Quantification of the mean organoid growth rate (μm/h) determined by live-cell imaging. (C) Mean organoid doubling time (h) calculated from live-cell imaging (*n* = 4–5); statistical analyses were performed using Kruskal Wallis test followed by Dunn's test for multiple comparisons test without significant differences *p* = 0.41 in (B) and *p* = 0.26 in (C).

## Discussion

The main objective of this study was to gain mechanistic insights into how alterations in microbial community composition and associated changes in the gut metabolome contribute to impaired IEB function and epithelial regeneration in obesity. We first metabolically phenotyped the rat cohort focusing on intestinal barrier alterations and inflammatory processes. Using colonic contents from rats fed either HFD or LFD, we showed that barrier impairing loss of epithelial regeneration and pro-inflammatory effects are, at least in part, mediated via intestinal luminal factors. A combined untargeted and targeted metabolomics approach was used to identify potential target metabolites that influence gut barrier integrity and to identify mechanisms behind gut barrier dysfunction in obesity. Detailed analysis identified UDP as a key metabolite mediating impaired epithelial regeneration through P2Y_6_ receptor signaling.

For *in vivo* studies, we compared male Wistar rats with diet-induced obesity (HFD) with a group on normal chow diet (LFD). Our findings, that in HFD-fed rats key junctional proteins like CLDN3 were reduced alongside increased levels of LPS indicate impaired intestinal barrier function and restructuring of intestinal barrier composition, as previously described.^55^ Unlike previous reports on obesity,^56^ ^,^ ^57^ we did not observe markers of systemic inflammation in the HFD group. However, increased levels of pro-inflammatory cytokines such as TNFα together with upregulated Tlr2 and Tlr4 expression indicate localized pro-inflammatory signaling in the colonic epithelium. The short 8-week HFD exposure suggests a time-dependent progression of inflammation during obesity. Early inflammatory responses may be confined to the gut before expanding systemically at later stages. This suggests distinct divergence between local mucosal inflammation and systemic low-grade inflammation, yet these processes are tightly interconnected. Evidence from diet-induced and genetic obesity models supports the notion that local gut-wall inflammation originates primarily at the epithelial–microbial interface.^58-60^ This concept is supported by our data of intestinal barrier dysregulation and a pro-inflammatory signature in transcriptome analysis of the colon. Based on our short-term model, we assume that local inflammatory signatures precede measurable systemic cytokine elevations. It must be emphasized that our current data do not provide direct in vivo evidence that impaired epithelial repair is causally linked to inflammation. Therefore, an hypothesis that will be evaluated in the future is that the gut may act as an early initiator of inflammatory responses in obesity.

Changes of gut microbiota and microbial metabolomes have been amply reported in obesity and in response to HFD.^61-63^ In our model we identified several genera as differentially abundant in the HFD group, however the taxa showing altered network correlations were notable suggesting that obesity was associated not only with changes in microbial composition but also with altered microbial interactions. Several of the taxa that were differentially correlated have known links to obesity and intestinal health. *Akkermansia* is widely regarded as beneficial for metabolic health and is negatively associated with obesity,^64^ while *Bifidobacterium* is known for its anti-inflammatory and weight-regulating properties.^65^ ^,^ ^66^ *Alistipes* has mixed associations with obesity, sometimes protective^67^ and sometimes pro-inflammatory.^68^ *Limosilactobacillus*, depending on strain, may have weight-modulating effects,^69^ further emphasizing the complexity of dietary influences on microbial interactions.

According to a previous study conducted in Caco2 cells, it has been demonstrated that intestinal luminal contents from HFD mice induce changes in epithelial barrier functions.^70^ Furthermore, data from our group have shown protective effects of intestinal luminal contents derived from rats who underwent RYGB surgery on the intestinal barrier in vitro.^25^ Possible barrier-impairing effects of contents from obese animals have not been studied in detail so far. In addition to Caco-2 cells, we also conducted our experiments in a more advanced model of murine organoids, better displaying the composition of the gut epithelium. For the first time, we show, that solutions derived from intestinal contents of HFD rats induce the same phenotype that was found in the colon of obese rats, including loss of CLDN3, and increased epithelial TNFα levels. Notably, colonic transcriptomics *in vivo* closely matched the transcriptomic response of epithelial cells exposed to HFD intestinal contents *in vitro*, indicating a shared molecular signature. Functionally, HFD-derived contents impaired epithelial regeneration, as demonstrated by reduced TEER and delayed scratch-wound closure. This led us to further focus on possible mechanistic effects of factors contained in the gut lumen on IEB regeneration in obesity.

We conducted LC-MS based metabolomics to analyze metabolites of the intestinal content solutions used in *in vitro* experiments. The untargeted metabolomics approach confirmed the distinct metabolomic profiles between the groups. Notably, most compounds that were present in one group, were in turn completely absent in the other group, underlining the deep metabolomic changes linked to a HFD and obesity. However, this approach failed to identify possible target compounds. This might be due to the fact, that the rat microbial metabolome has not been studied extensively yet, leaving many unknown compounds. Further studies are needed to identify metabolomic profiles in rats to better assess the translational capacities of these animal models in the metabolomic context.

Nonetheless, the targeted metabolomics approach showed a similar picture compared to the untargeted approach, with very distinct colonic metabolomic profiles between the groups. As compounds of interest, we identified several uridine-metabolites, with especially UDP-Glc and UDP being of interest. While the variance is very high in the HFD group, resulting in statistically not significant results, the fact that several compounds linked to uridine are increased suggests a certain effect of the HFD on these metabolites. Increased uridine metabolites may originate from the intestinal epithelium due to altered cell turnover, from the gut microbiota through peptidoglycan or polysaccharide biosynthesis, or from host systemic uridine metabolism via hepatobiliary circulation. In addition, although the quantitative contribution remains unknown microbial cell-wall remodeling and lysis may release UDP-sugar intermediates. Dietary nucleotide intake and increased host glycosylation demands may further contribute to the intestinal uridine pool.^71^ ^,^ ^72^ Because UDP and uridine are embedded in both host and microbial metabolic networks, future work will have to distinguish among these sources using compartment-specific methods.

According to experiments carried out with UDP-Glc and UDP alone we identified UDP as a potential mediator of impaired epithelial regeneration leading to reduced IEB function as a consequence of HFD using cell culture models and intestinal epithelial organoid models. This was further substantiated by the observation that the specific inhibitor of UDP receptor P2Y_6_ abolished the negative effects of UDP on epithelial regeneration. A role of P2Y_6_-induced modulation of epithelial regeneration by UDP has been recognized earlier in a study, where UDP appeared to have protective effects on intestinal regeneration.^73^ ^,^ ^74^ On the other hand P2Y_6_ activation has been linked to intestinal inflammation and has been shown to be causative for impaired epithelial regeneration in various models.^53^ ^,^ ^54^ Our data at epithelial cell level provide evidence that both HFD-derived UDP and UDP alone impair epithelial regeneration. The fact that ERK inhibition partially attenuated HFD-induced effects on epithelial regeneration and P2Y_6_, combined with the observation that P2Y_6_ activation triggers ERK/MAPK signaling, underlines this assumption at a functional level. This is further supported by gene set enrichment analysis of ERK/MAPK signaling in the colon of HFD-fed rats and in epithelial cells subjected to HFD contents *in vitro*. More detailed studies will be required to resolve the precise mechanisms underlying P2Y_6_-dependent epithelial repair.

However, direct human data validating the role of UDP and P2Y_6_ signaling in obesity-related intestinal dysfunction are very limited. Translational relevance of our observations can be assumed from the presence of the P2 purinergic receptors throughout the gastrointestinal tract.^75^ Direct measurements of intracolonic UDP in obese humans have not yet been performed but the need for such studies has been highlighted earlier.^72^ ^,^ ^76^ Nonetheless, human studies have implicated purinergic signaling, particularly through P2Y receptors, in regulating intestinal barrier integrity. Activation of P2Y_6_ receptors has been shown to promote cytoskeletal remodeling and increase permeability in human intestinal epithelial monolayers exposed to Clostridium difficile toxins. Notably, P2Y_6_ blockade attenuated toxin-induced barrier dysfunction and inflammatory responses, providing mechanistic support for our in vitro findings. These results suggest that P2Y_6_-targeted strategies may be relevant in modulating intestinal barrier function in human conditions, including those associated with obesity.^54^ Further support for a pathogenic role of P2Y_6_ receptor signaling as an important mediator of intestinal inflammation is derived from NTPDase8-deficient mice which is the dominant receptor for the hydrolysis of nucleotides. Loss of this enzyme resulted in exacerbation of experimental colitis by activation of P2Y_6_ receptor.^77^

In summary, our current data show that HFD-associated changes in gut microbiota and metabolite composition contribute directly to impaired intestinal barrier function, with local mucosal inflammation appearing before systemic inflammatory changes. Intestinal contents from HFD-fed rats reproduced the barrier defects *in vivo* and regenerative impairment in epithelial models *in vitro*. Our current findings identify UDP–P2Y_6_ receptor signaling as a mechanistic link between HFD, local inflammation, and defective epithelial repair in early obesity. These insights lay the groundwork for future cohort studies aimed at translating our findings into clinical settings.

## Supplementary Material

Supplementary material Suppl figure S1_revised Kopie.jpg

Supplementary material Suppl figure S3_revised Kopie.jpg

Supplementary material Suppl_figure_S4_resize 1.jpg

Suppl figure S2_revised Kopie.jpg Suppl figure S2_revised Kopie.jpg

Supplementary material Supplementary Material.docx

## Data Availability

There are no restrictions on data availability. The supporting data values are available in supplemental material. Additional data are available by request to the corresponding author. All data and study materials are available on GEO under the accessions GSE302243 (colonic tissue), GSE302244 (organoids) and GSE302254 (microbiome). The analysis pipeline for the organoid growth pattern is available from the corresponding author on request.
