# Supplementary material for: High-fat diet-derived metabolite uridine diphosphate (UDP) impairs epithelial regeneration by P2Y 6 -receptor signaling
Source: Gut Microbes. 2026 Oct 15;18(1):2748160. doi: 10.1080/19490976.2026.2748160 (PMC13682407; doi:10.1080/19490976.2026.2748160)
Supplement: Supplementary file 1 [file KGMI_A_2748160_SM7375.docx]

**Supplemental Material**

**Supplemental figure S1**

Cytokine levels in systemic (upper plots) and portal vein (lower plots) serum in LFD and HFD rats. Boxplots show the median (horizontal line), n = 4-6, no significant differences were detected, as determined by unpaired t-test.

**Supplemental figure S2**

A TEER of differentiated murine organoids treated with different concentrations of UDP-Glucose. B Representative immunoblot bands of ERK signalling, CLDN3, TNFα and Caspase in murine organoids treated with UDP-Glucose.

**Supplemental figure S3**

A Representative images of wound healing assays in 2D monolayers of murine organoids treated with LFD or HFD content and FR180204. B Quantification of wound healing expressed as Area of scratch relative to control. C Representative wound healing assays of 2D monolayers of murine organoids and quantifications under control conditions and after DMSO application to exclude DMSO effects.

**S5 Supplemental figure S4**

A Representative micrographs of the same 3D organoid cultures are shown at the start of the experiment (0 h) and after 24, 48, and 72 h under control conditions and after incubation with MRS 2578; quantifications are presented in figure 8B,C.
